# Hypertension Management in Brazil: Usual Practice in Primary Care—A Meta-Analysis

**DOI:** 10.1155/2017/1274168

**Published:** 2017-07-02

**Authors:** Rafael V. Picon, Juvenal S. Dias-da-Costa, Flavio D. Fuchs, Maria Teresa A. Olinto, Niteesh K. Choudhry, Sandra C. Fuchs

**Affiliations:** ^1^Postgraduate Studies Program in Cardiology, School of Medicine, Universidade Federal do Rio Grande do Sul, R. Ramiro Barcelos 2600, 90035-003 Porto Alegre, RS, Brazil; ^2^Postgraduate Program of Collective Health, Universidade do Vale do Rio dos Sinos, São Leopoldo, RS, Brazil; ^3^Division of Cardiology, Hospital de Clinicas de Porto Alegre, Universidade Federal do Rio Grande do Sul, Porto Alegre, RS, Brazil; ^4^Division of Pharmacoepidemiology and Pharmacoeconomics, Brigham and Women's Hospital, Harvard School of Medicine, Harvard University, Boston, MA, USA; ^5^Postgraduate Studies Program in Epidemiology, School of Medicine, Universidade Federal do Rio Grande do Sul, R. Ramiro Barcelos 2600, 90035-003 Porto Alegre, RS, Brazil

## Abstract

Knowing the usual clinical practice is relevant for evaluations in health care and economic policies of management of hypertension. This study aimed to describe the usual management of hypertension in the Brazilian primary healthcare system through a systematic review and meta-analysis. The search of population-based studies conducted in Brazil was undertaken using PubMed, EMBASE, and Brazilian databases. Eligible studies were those conducted in adults with hypertension (blood pressure (BP) ≥ 140/90 mmHg or using BP lowering drugs). Three datasets' data were analyzed: SESI study (in Brazilian workers); HIPERDIA (Brazilian Registration and Monitoring of Hypertensive and Diabetic Patients Program); and a population-based study. Meta-analysis has been performed using the fixed and random effect models. A total of 11 studies or data sets were included in the systematic review. Hypertensive individuals had, on average, 2.6 medical visits annually and 18.2% were on diuretics (*n* = 811 hypertensive patients) and 16.2% on ACE inhibitors (*n* = 1768 hypertensive patients). BP control rate ranged from 43.7 to 67.5%; 35.5% had measured total cholesterol and 36.5% determined fasting plasma glucose in the previous 12 months. Thiazide diuretics and ACE inhibitors were the most used BP lowering medications as single drugs, but the control rate of hypertension is insufficient.

## 1. Introduction

Assessment of costs and health outcomes can generate estimates to compare and choose among screening, diagnostic, or therapeutic strategies, which should be incorporated into the healthcare system. The endpoint is to achieve maximum health gains with the available resources, respecting the expectations of the population covered by the healthcare system and considering the limited resources [1]. Health economic evaluations (HEES) are useful tools for managers and policy makers to choose the best allocation of available resources, or even about the assimilation of a new health technology. HEES are particularly useful to decide on incorporation of new strategies for prevention or control of noncommunicable diseases. In Brazil, the public health system,* Sistema Único de Saúde* (SUS), is based on universal free access to healthcare to the whole population, with decentralization provided at all levels, from prevention to high complexity level, shared by federal, state, and municipal government [2]. Blood pressure lowering medication are available at the SUS, freely distributed, and a list of drugs include thiazide diuretic, beta-blocker, ACE inhibitor, and an angiotensin receptor blocker (ARB). Health plan physicians are an alternative placed between the public and private systems, ranging from partial to full coverage, and usually with no refund for costs with blood pressure lowering agents. HEE is necessary to decide whether new drugs will be made available by the SUS.

It is estimated that hypertension (HT) affects 28.7% (95% CI: 26.2–31.4%) of the Brazilian adult population, with decreasing temporal trend in the rate of hypertension control [9]. In a meta-analysis of population-based studies, the control rate was 25% [9]. HT is responsible for significant global morbidity and mortality [10]. However, there is no systematic analysis of economic costs for diagnosis, risk stratification, and treatment of HT in Brazil [11].

A suitable HEE should include the comparison between the strategy of the Brazilian Guidelines of Hypertension [12] and the usual practice (status-quo), aiming at the primary prevention of CVD in patients of primary care. The status-quo can serve as a baseline for comparisons whenever a new health strategy is being considered for implementation [13]. Hence, our study aimed to describe the usual practice, in the context of primary care of the SUS in Brazil, regarding the diagnosis, risk stratification, and pharmacological treatment of HT.

## 2. Methods

### 2.1. Design and Data Sources

Studies conducted at population-based or outpatient, cross-sectional, or cohort studies, carried out since 2000, were searched in the databases of PubMed, EMBASE, and population-based databases of the Brazilian Virtual Health Library (VHL; http://brasil.bvs.br/en/). The following search strategies were used: VHL using Descriptors in Health Sciences: “Hipertensão” AND “Atenção Primária à Saúde” AND “Brasil”; on EMBASE using entrees: “brazil”/exp AND “hypertension”/exp AND “primary health care”/exp; and on PubMed using MeSH Terms: ((“Hypertension”[Mesh]) AND “Primary Health Care”[Mesh]) AND “Brazil”[Mesh]. We also evaluated articles, which were included in a meta-analysis of the prevalence of hypertension [9], conducted by the authors. Articles that met the eligibility criteria were included: population-based cross-sectional or cohort studies performed in participants with 18 years or more, between 1980 and 2010. We also incorporated in meta-analysis four sets of data available to us, regardless of the systematic review searches [14–16].

### 2.2. Eligibility Criteria for the Review and Screening Process

Studies conducted in Brazil including patients with HT treated in any primary care facility affiliated to the SUS or population-based studies, which reported data in adults with HT were considered eligible for data extraction. Hospitalized patients were outside of the scope of this analysis and were excluded. The search results were handled in a double-screening process: their titles and abstracts were scrutinized and those eligible had their full-texts examined. Duplicated results were excluded. Studies conducted in pregnant women were excluded.

### 2.3. Target Population and Variables of Interest

The target population consisted of Brazilian adults (≥18 years) who either had blood pressure (BP) ≥ 140/90 mmHg or were on treatment with BP lowering agents (BPLM), enrolled from the general population or among those who were undertaking treatment in primary care settings. In order to collect information on management of patients and ensure comparability with the Brazilian Guidelines of Arterial Hypertension [12] and among guidelines [17], the following variables have been extracted: frequency of medical consultations and type of health insurance (e.g., SUS, private health plan) used most of the time, number and frequency of diagnostic tests suggested by this guideline (e.g., ECG, fasting glucose, and chest X-ray), antihypertensive classes, clinical characteristics, such as systolic BP (SBP), total and high-density lipoprotein cholesterol (HDL), and prevalence of other relevant comorbidities (diabetes mellitus (DM) and smoking) [18]. The rates of smoking and prevalence of DM among hypertensive individuals enrolled in the HIPERDIA (Brazilian Registration and Monitoring of Hypertensive and Diabetic Patients Program), from May 2002 to April 2012, were also evaluated [5].

### 2.4. Data Analysis

Continuous variables with normal distribution were presented as means and standard deviations (SD). Binary data were presented as proportions using point estimates and 95% confidence interval (95% CI). Meta-analyses were performed with the pooling of means or proportions: when the same variable of interest was measured using the same method across studies (e.g., self-reported diabetes versus fasting plasma glucose) and the same, or interchangeable, metric (e.g., one laboratory test per month equals 12 tests per year). Random effects model was mostly used; however, fixed effect model was employed when nonsignificant heterogeneity (*p* value ≥ 0.05) was observed, as measured by Cochran's *Q*. The *I*^2^ statistic was employed as a continuous measure of heterogeneity. Statistical analysis was performed in the Statistical Package for the Social Science (SPSS; version 17.0, IL, USA) and meta-analysis using Comprehensive Meta-Analysis (software version 2.0; Biostat, Englewood, NJ). Since this study is entirely descriptive, we did not formulate nor test any hypothesis.

## 3. Results

**Figure 1:**
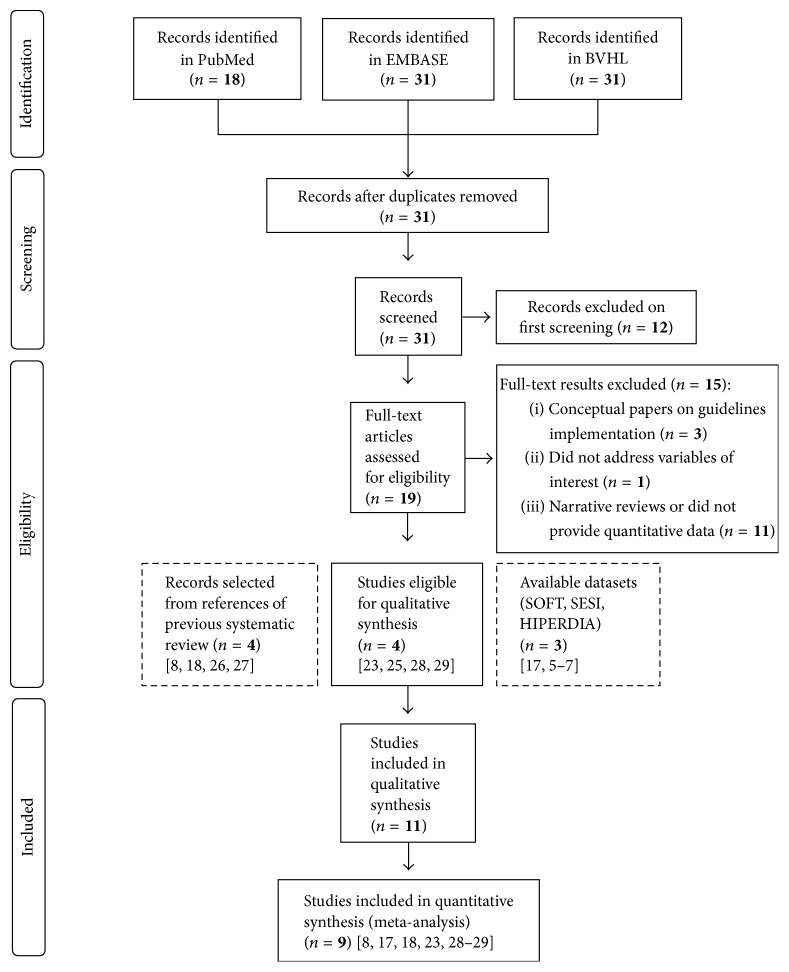
Flow chart of records retrieved, screened, and included in the systematic review.

Figure 1 depicts the flow of search results in this review. The VHL search retrieved 31 results, PubMed 18 results, and EMBASE another 31 results. EMBASE and VHL retrieved identical results, which encompassed all the 18 articles found in PubMed. After the first screening and removal of overlapping result across databases, 19 articles were deemed eligible. Four studies reported data on at least one of the studied variables and were included in the meta-analysis. There were three available datasets: (i) two datasets from population-based cross-sectional studies conducted in large representative samples of two cities from Southern Brazil, Porto Alegre (capital of the state; SOFT study; *n* = 1858) [6, 7]; (ii) a third dataset was originated from a nationwide cross-sectional study conducted among 1148 industry workers (SESI study previously carried out by one of the authors) [3, 4]; (iii) a nationwide registry of hypertensive and diabetic patients treated in primary care of SUS (with of 7.3 million individuals on treatment for hypertension) collected from the HIPERDIA [5]. Another four studies included in a previous systematic review were considered eligible and, hence, were added to this meta-analysis along with the three aforementioned datasets, rendering a total of 11 studies [8, 19–21].

Table 1 shows characteristics of the studies and HIPERDIA registry that provided data on clinical characteristics of individuals with HT according to sex. Studies that have data on mean systolic blood pressure and blood pressure control are presented in Table 1 as well. In the SESI study, men and women had similar SBP, but women had higher rate of hypertension control than men. In the population-based studies men and women had similar mean systolic blood pressure and rates of hypertension control. Higher prevalence of DM was observed among patients registered in HIPERDIA, especially in comparison to the participants of the SESI study. Smoking prevalence rates were more evenly distributed across the SESI study, HIPERDIA registry, and population-based studies, with higher prevalence of current smokers in men in comparison to women.

Table 2 presents information from seven studies that provided data on pharmacotherapy, diagnostic tests, and medical appointments. Less than half of the hypertensive subjects were using a single BPLM, and the most common class of BPLM was thiazide diuretics, followed by angiotensin-converting enzyme (ACE) inhibitors. Thiazide diuretics combined with ACE inhibitors were the most frequent 2-drug combination in use (14.9%), followed by thiazides and beta-blockers (9.4%).

**Figure 2:**
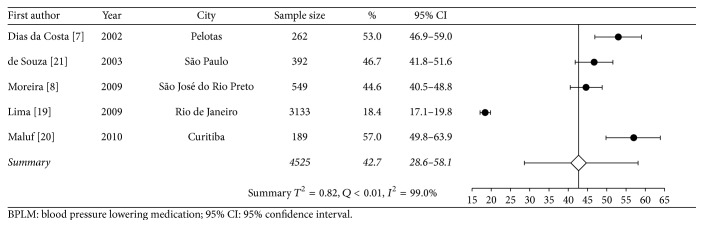
Meta-analysis of proportion of use of one BPLM (in chronological order according to the data collection year).

**Figure 3:**
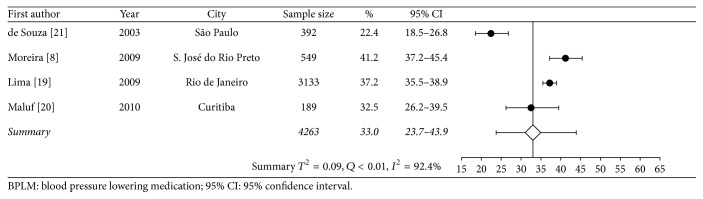
Meta-analysis of proportion of use of two blood pressure lowering medication.

**Figure 4:**
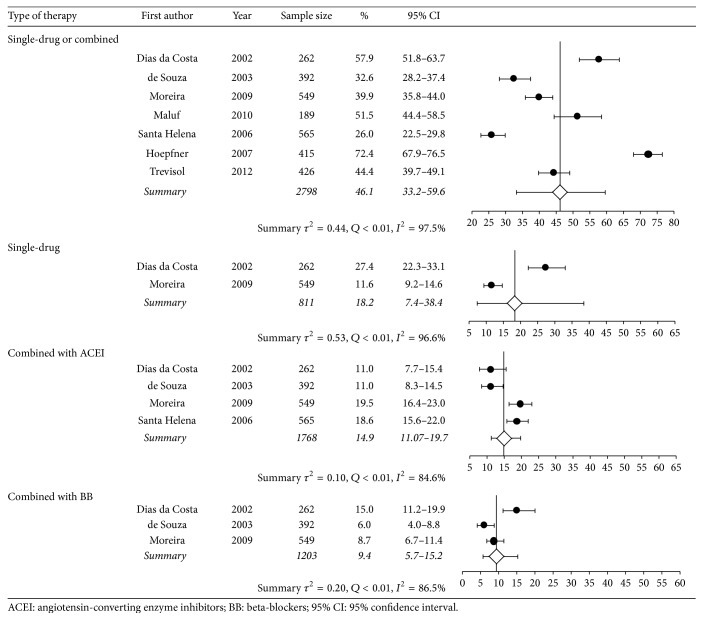
Meta-analysis of proportion of use of thiazide-based therapy.

Data on diagnostic tests came from one study, and estimates on most used type of medical services came from another study. Approximately a third of individuals with known HT had fasting plasma glucose, serum triglycerides, total cholesterol, and creatinine level measured in the previous 12 months. On average, an adult with HT had 2.6 medical appointments per year, and more than half of subjects who sought medical appointments used mostly those provided by the SUS. Figures 2, 3, and 4 are forest plots illustrating contents from Table 2.  Figure 2 shows considerable heterogeneity among studies, mostly due to the study of Lima et al. [19], carried out in Rio de Janeiro.

## 4. Discussion

This systematic review examined all available databases that provide information on the management of HT among the Brazilian adult population. Some of them described the compliance with treatment guidelines of the State Health Department or provided an opinion on this matter [22, 23]. One article assessed physicians' compliance with HT treatment according to a Municipal Health Department guideline and detected noncompliance rates of 56.8%, 63.8%, and 54.0% regarding HT staging, cardiovascular risk classification, and choice of treatment, respectively [20].

Official data estimate that about 75% of the country's population depends exclusively on SUS for health care [24], but it has not been confirmed by the information from a population-based study carried out in the city of Pelotas [7]. The divergence may be explained by at least two biases from both sources of information: (i) the use of SUS services probably does not reach the 75% rate for all health conditions; (ii) recall bias of the medical appointments made in the previous month by participants of the population-based study. Nonetheless, the systematic review data derives from a single city; therefore it is hardly representative of the entire country.

Medical treatment by use of blood pressure lowering agents, conversely, was more often reported, so HT treatment data might be more representative of nationwide clinical practice within the SUS. Blood pressure control rates among individuals with HT could not be summarized through the results of all included studies, but it was reported for three population-based studies. The rates of controlled hypertension were higher than that reported in a previously systematic review, which identified a pooled control estimate of 24.1% (10.1–47.3%) [9]. The use of thiazide-type diuretics (as single or combined-drug therapy) was widespread in our analysis. This is in concordance with the best available evidence of effectiveness and current guidelines that advocate for the use of these drugs as first line treatment [12]. However, considering that there are few absolute contraindications for the use of diuretics, one may argue that the 41% usage rate should have been higher. On the other side, thiazide diuretics are more often used in Brazil than in Denmark, Finland, Germany, Norway, Sweden, Netherlands [25], Portugal [26], Spain [27], Mexico [28], and the United States [29]. Among other antihypertensive drugs, the use of beta-blockers was similar to that reported in other countries, but the prevalence of calcium channel blockers (CCB), ACE inhibitors, and ARBs use was much lower. CCB and ARB were rarely used to treat HT, since these drugs were not available in the SUS by the time that most of the studies were conducted. An ARB agent, by the opposite, was recently incorporated in the SUS.

Although there are several population-based studies that evaluated the prevalence of HT all over the country, only four studies provided information on how participants with hypertension were treated. The vast majority of these field studies restricted their assessment to measuring the prevalence of HT and other diseases among the general population, reporting little or no information with regard to individuals with HT (mean age of participants with HT, blood pressure control rates, etc.). Five studies reported the prevalence of hypertensive patients using one BPLM. The summary estimate of prevalence was reduced due to the weight of a study performed at a primary care center in Rio de Janeiro [19]. This study introduced heterogeneity due to the variability in the number of follow-up visits, ranging from 1 to 27, and to participants who did not use any medication (7.8%) and used two or more BPLMs (73.8%) and 18.4% who used only one drug to decrease BP. This study had a major contribution to the overall results.

Another limitation of the present study is the overall paucity of publications. This scarcity contrasts with a huge amount of data on patient management generated every day inside the SUS [2], with more than 40.4 million medical appointments recorded in the HIPERDIA registry from August 2011 to July 2012 [5].

Finally, our data did not cover the years since the new public program, Farmácia popular (popular pharmacy), for delivery of drugs and devices was launched, including the subsidized offering of losartan (90% of rebate). Twenty percent of the population is presently covered (http://www.brasil.gov.br/saude/2016/03/aqui-tem-farmacia-popular-atende-38-milhoes-de-brasileiros-em-10-anos, accessed in December, 22, 2016). The impact of this program in the use of BP lowering drugs was not captured in the surveys available for this systematic review.

## 5. Conclusions

Despite these limitations, the estimates presented by this review are the best available evidence about the pattern of use of BP lowering drugs and rate of BP control in Brazil. Further studies should prospectively collect data to better describe the impact of new BP lowering agents. Thiazide diuretics and ACE inhibitors were the most frequently used as a single drug, and ACE inhibitors were the BP lowering medications more frequently prescribed, but the rate of hypertension control is insufficient.

## Figures and Tables

**Table 1:** Clinical characteristics of individuals with hypertension from the SESI study, the HIPERDIA registry, and from meta-analyses of four population-based studies.

Studies and clinical characteristics	Mean (±SD)/prevalence (95% CI)	
	Men	Women
*SESI study [3, 4]: participants *(*n*)	1034	114
Age (years)	40.6 (11.8)	41.5 (9.2)
Systolic blood pressure (mmHg)	152.5 (15.9)	150.4 (21.7)
Controlled blood pressure	31.8 (26.3–37.7)	56.9 (45.5–67.7)
Total cholesterol (mg/dL)	188.8 (40.0)	196.0 (39.2)
HDL cholesterol (mg/dL)	49.3 (20.9)	56.8 (12.7)
Current smokers	18.3 (15.9–20.7)	15.5 (8.9–22.1)
Diabetes mellitus (DM)	5.6 (4.2–7.0)	3.5 (0.1–6.9)
*HIPERDIA registry [5]: participants *(*n*)	2.5 million	4.8 million
Current smokers	21.42 (20.4–22.5)	15.6 (14.7–16.5)
Diabetes mellitus	21.9 (19.8–24.0)	24.9 (22.9–27.0)
Current smokers with DM	6.3 (5.8–6.8)	5.6 (5.2–6.0)
*Population-based studies: participants *(*n*)	5064	8126
Systolic blood pressure (mm Hg)		
Trevisol et al. [6]	146.8 (20.7)	138.9 (22.6)
Dias da Costa et al. [7]	144.5 (8.3)	146.1 (20.7)
Controlled blood pressure		
Trevisol et al. [6]	47.8 (39.7–56.1)	43.7 (38.5–49.2)
Dias da Costa et al. [7]	65.7 (53.7–75.9)	67.5 (60.7–73.7)
Moreira et al. [8]	53.0 (46.9–58.4)	52.7 (45.6–58.9)
Current smokers	21.7 (17.2–27.0)^‡^	14.8 (10.1–21.0)^*∗*^
Diabetes mellitus^*∗∗*^	13.5 (12.5–14.4)^†^	13.2 (8.3–20.7)^††^

^‡^ *Q*  *p* < 0.01; *I*^2^ = 90.0%. ^*∗*^Data from 7867 women. Q *p* < 0.01; *I*^2^ = 94.1%. ^†^Fixed effect model analysis. *Q*  *p* = 0.24; *I*^2^ = 27.4%. ^*∗∗*^Data from 4912 men and 7867 women; ^††^*Q*  *p* < 0.01; *I*^2^ = 87.3%.

**Table 2:** Frequency of blood pressure lowering medication use, diagnostic tests, and medical appointments among hypertensive subjects.

	Prevalence (95% CI)/mean (±SD)	Heterogeneity	
	*Status quo*	*Q* *p* value	*I* ^2^
*Blood pressure lowering medication (%)*			
In use of one BPLM	42.7 (28.6–58.1)	<0.01	99.0
In use of two BPLM	33.0 (23.7–43.9)	<0.01	92.4
*Type of BPLM *			
Thiazide diuretics			
Single-drug therapy or combined with another drug	41.1 (26.4–57.6)	<0.01	98.3
Single-drug therapy	18.2 (7.4–38.4)	<0.01	96.6
Combined with ACEI	14.9 (11.1–19.8)	<0.01	84.6
Combined with BB	9.4 (5.7–15.2)	<0.01	86.5
Combined with CCB^‡^	5.0 (2.4–7.6)	NA	NA
Angiotensin-converting enzyme inhibitors (ACEI)			
Single-drug therapy or combined with another drug	41.1 (20.2–65.7)	<0.01	97.7
Single-drug therapy	16.2 (11.6–22.1)	<0.01	85.0
Combined with BB^†^	3.4 (2.5–4.7)	0.07	62.1
Combined with CCB^‡^	4.0 (2.1–5.9)	NA	NA
Beta-blockers (BB)			
Single-drug therapy or combined with other BPLM	21.2 (17.3–25.8)	<0.01	84.3
Single-drug therapy^†^	10.0 (8.1–12.3)	0.17	46.2
Combined with CCB^‡^	2.3 (0.5–4.1)	NA	NA
Calcium channel blockers (CCB)			
Single-drug therapy or combined with other BPLM	10.0 (7.5–13.3)	<0.01	72.4
Single-drug therapy^‡^	3.9 (1.6–6.2)	NA	NA
Angiotensin receptor blockers (ARB)			
Single-drug therapy or combined with other BPLM^†^	2.3 (1.4–3.6)	0.06	71.2
*Diagnostic tests and procedures (%) among hypertensive subjects*			
Previous month testing			
Electrocardiography^‡^	6.3 (3.9–8.8)	NA	NA
Any radiography^‡^	9.7 (6.8–12.7)	NA	NA
Any urine test^‡^	8.4 (5.6–11.2)	NA	NA
Any blood test^‡^	12.6 (9.3–16.0)	NA	NA
Direct ophthalmoscopy^‡*∗*^	35.0 (30.2–39.8)	NA	NA
Previous 12-month testing			
Serum potassium^‡^	19.5 (13.9–25.2)	NA	NA
Serum creatinine^‡^	31.0 (24.4–29.6)	NA	NA
Total serum cholesterol^‡^	35.5 (28.7–42.3)	NA	NA
Serum LDL or HDL cholesterol^‡^	25.0 (18.3–31.2)	NA	NA
Serum triglycerides^‡^	34.0 (27.3–40.8)	NA	NA
Fasting plasma glucose^‡^	36.5 (29.6–43.4)	NA	NA
Urine analysis^‡^	25.0 (18.8–31.2)	NA	NA
Medical appointments (%) among hypertensive subjects			
Annual mean of medical appointments	2.62 (2.37)	0.5	0
Mostly using Brazilian Health Care System^‡^	51.2 (46.1–56.2)	NA	NA
Mostly using private physicians^‡^	20.9 (16.8–25.1)	NA	NA
Mostly using health plan physician^‡^	13.0 (9.6–16.4)	NA	NA
Mostly using emergency services^‡^	1.9 (0.5–3.2)	NA	NA
Others^‡^	13.0 (9.6–16.4)	NA	NA

^†^Fixed effect analysis. ^‡^Based on one study. ^*∗*^Since the diagnosis of hypertension; LDL: low-density lipoprotein; HDL: high-density lipoprotein; *Q*  *p* value and *I*^2^–: nonapplicable; that is, only one study provided data; NA: not applicable.
